# Effects of Liraglutide, Empagliflozin and Their Combination on Left Atrial Strain and Arterial Function

**DOI:** 10.3390/medicina60030395

**Published:** 2024-02-26

**Authors:** Konstantinos Katogiannis, John Thymis, Foteini Kousathana, George Pavlidis, Emmanouil Korakas, Aikaterini Kountouri, Konstantinos Balampanis, Vasiliki Prentza, Gavriella Kostelli, Helen Michalopoulou, Damianos Tsilivarakis, Vaia Lambadiari, Ignatios Ikonomidis

**Affiliations:** 1 Second Cardiology Department, Attikon University Hospital, Medical School, National and Kapodistrian University of Athens, 15772 Athens, Greece; johnythg@gmail.com (J.T.); kosteligavriela@hotmail.com (G.K.); elenimixa91@gmail.com (H.M.); tsilyd@yahoo.com (D.T.); ignoik@gmail.com (I.I.); 2 Second Department of Internal Medicine, Attikon University Hospital, Medical School, National and Kapodistrian University of Athens, 15772 Athens, Greece; f.kousathana@hotmail.com (F.K.); geo_pavlidis@yahoo.gr (G.P.); mankor-th@hotmail.com (E.K.); katerinak90@hotmail.com (A.K.); kostasbalabanis@gmail.com (K.B.); prentzavasiliki@gmail.com (V.P.); vlambad@otenet.gr (V.L.)

**Keywords:** speckle tracking echocardiography, left atrium, Diabetes mellitus, SGLT-2i, GLPI-1RA, heart failure with preserved ejection fraction, atrial mechanics, atrial function

## Abstract

*Background and Objectives*: Glucagon-like peptide-1 receptor agonists (GLP-1RA) and sodium-glucose cotransporter-2 inhibitors (SGLT-2i) are cardioprotective drugs. We investigated their effects on left atrial function, a major determinant of cardiac diastolic dysfunction in type 2 diabetes mellitus. We also explored the association of changes in arterial stiffness with those of the LA strain after treatment. *Materials and Methods*: A total of 200 patients (59.5 ± 9.1 year old, 151 male) with type 2 diabetes mellitus treated with metformin were randomized to insulin (n = 50 served as controls), liraglutide (n = 50), empagliflozin (n = 50) or their combination (liraglutide + empagliflozin) (n = 50). We measured at baseline and 6 months post-treatment: (a) left atrial and global left ventricular longitudinal strain by speckle tracking echocardiography; (b) pulse wave velocity (PWV) and central systolic blood pressure. *Results*: At baseline, there was a correlation of the LA reservoir strain with PWV (r = −0.209, *p* = 0.008), central SBP (r = −0.151, *p* = 0.030), EF (r = 0.214, *p* = 0.004) and GLS (r = −0.279, *p* = 0.009). The LA reservoir change 6 months post-treatment was correlated with the PWV change in all groups (r = −0.242, *p* = 0.028). The LA reservoir change 6 months post-treatment was correlated with the GLS change in all groups (r = −0.322, *p* = 0.004). Six months after intervention, patients treated with liraglutide, empagliflozin and their combination improved the left atrial reservoir strain (GLP1RA 30.7 ± 9.3 vs. 33.9 ± 9.7%, *p* = 0.011, SGLT2i 30 ± 8.3 vs. 32.3 ± 7.3%, *p* = 0.04, GLP1&SGLT2i 29.1 ± 8.7 vs. 31.3 ± 8.2, *p* = 0.007) compared to those treated with insulin (33 ± 8.3% vs. 32.8 ± 7.4, *p* = 0.829). Also, patients treated with liraglutide and the combination liraglutide and empagliflozin had improved left atrial conduction strain (*p* < 0.05). Empagliflozin or the combination liraglutide and empagliflozin showed a greater decrease of PWV and central and brachial systolic blood pressure than insulin or GLP-1RA. (*p* < 0.05). *Conclusions*: Impaired aortic elastic properties are associated with a decreased LA strain in type 2 diabetics. Treatment with liraglutide, empagliflozin and their combination for 6 months showed a greater improvement of left atrial function compared to insulin treatment in parallel with the improvement of arterial and myocardial functions.

## 1. Introduction

Diabetes mellitus (DM) is known for its detrimental effects on vascular endothelium and myocardium [1]. Left ventricular (LV) function may be impaired in patients suffering from DM [2] despite a normal LV ejection fraction, resulting in symptomatic heart failure [3].

Moreover, medications, such as glucagon-like peptide-1 receptor agonists (GLP-1 RA) and sodium-glucose co-transporter-2 inhibitors (SGLT-2i), have been included in the evidence-based treatment of DM, as they provide well documented cardioprotective effects [4,5,6]. In particular, sodium-glucose co-transporter-2 (SGLT-2) inhibitors, classified as antihyperglycemic agents, target the SGLT-2 proteins found in the proximal convoluted tubules. Their mechanism of action involves inhibiting the reuptake of glucose that has been filtered in the tubular lumen. All four FDA-approved SGLT-2 inhibitors—canagliflozin, dapagliflozin, empagliflozin and ertugliflozin—act by reducing the reabsorption of filtered glucose, lowering the renal threshold for glucose and facilitating urinary glucose excretion. These inhibitors contribute to a decrease in HbA1c by approximately 0.7% [7].

Data from recent metanalyses support the following indications for SGLT-2i prescription: (i) enhancing glycemic control in patients with type 2 DM, (ii) mitigating major adverse cardiovascular events (nonfatal myocardial infarction, nonfatal stroke, cardiovascular death) in patients with type 2 DM and a history of cardiovascular disease, (iii) decreasing the peril of cardiovascular admission and death for heart failure in patients with heart failure with reduced ejection fraction (HFrEF) in New York Heart Association (NYHA) class II–IV, (iv) reducing the risk of renal function deterioration and admission in patients with chronic kidney disease and (v) improving cardiovascular results in patients with heart failure with preserved ejection fraction (HFpEF) [7,8,9]. On the other hand, medications known as glucagon-like peptide-1 (GLP-1) agonists, also referred to as GLP-1 receptor agonists, incretin mimetics or GLP-1 analogs, are used to manage type 2 diabetes mellitus and, in some instances, obesity. The most common drugs included in this category are liraglutide, albiglutide, dulaglutide, semaglutide, exenatide and lixisenatide. While metformin is the preferred initial treatment for type 2 diabetes according to the American Diabetes Association, the consideration of adding a GLP-1 analog is recommended for patients with inadequate diabetes control, indicated by an HbA1c level exceeding 1.5% above the target. This recommendation is especially pertinent for patients dealing with atherosclerosis, heart failure or chronic kidney disease [9,10].

Both GLP1-RA and SGLT2i are effective in reducing atherosclerotic MACE in patients with established atherosclerotic cardiovascular disease. However, SGLT2i additionally demonstrate benefits such as preventing hospitalization for heart failure and reducing the estimated glomerular filtration rate across a broad range of patients [8].

Echocardiography is a contemporary method that offers a thorough analysis of cardiac function [11]. Speckle tracking echocardiography (STE) offers a detailed analysis of LV [12], right ventricular (RV) [13] and left atrial (LA) myocardial deformation [14]. Thus, longitudinal strain assessed by STE is a sensitive marker for detecting subtle myocardial dysfunction. Myocardial longitudinal deformation plays a critical role in the clinical assessment of various cardiac conditions, contributing to early diagnosis, risk stratification and the management of patients with cardiovascular disease. Its clinical implications include the identification of ischemic heart disease, assessment of cardiomyopathies, monitoring of heart failure, evaluation of valvular heart disease and early detection of cardiotoxicity in patients undergoing chemotherapy [12]. In particular, the LA strain has shown an independent and additive value for the diagnosis of LV diastolic dysfunction and prediction of adverse cardiac events in all ranges of LV ejection fraction over conventional echocardiographic parameters [15].

Moreover, in patients with DM, impaired endothelial glycocalyx and increased arterial stiffness, as assessed by pulse wave velocity (PWV), are associated with impaired LV myocardial deformation [16,17].

A twelve-month treatment with GLP-1RA, SGLT-2i or their combination showed a greater improvement in endothelial glycocalyx and PWV in parallel with an improvement in LV myocardial deformation and effective cardiac work than insulin treatment in patients with DM type 2 [17]. An improvement in peripheral arterial stiffness, as defined by a decrease in PWV, leads to a reduced LV afterload and enhances LV deformation. This has an impact on arterio–ventricular coupling and ameliorates LV function. Thus, LV end diastolic pressure declines and LV diastolic properties are improved. As a result, LA function may be facilitated, and this can be reflected by an increase in LA deformation [18,19].

We hypothesized that treatment with GLP-1RA (liraglutide), SGLT-2i (empagliflozin) or their combination may improve the LA strain, to a greater extent than the traditional insulin plus metformin regimen. Thus, we examined the change in the LA strain, as assessed by STE, at baseline and after 6 months of treatment in four parallel groups of patients with DM type 2 treated with metformin and insulin, liraglutide, empagliflozin or the combination, respectively. We also explored the association of changes in arterial stiffness with those of the LA strain after treatment.

## 2. Patients and Methods

### Patients

We evaluated 238 patients with type 2 DM in order to be included in the study. Patients were assessed at the cardiometabolic outpatient facility of Attikon University Hospital. Patients could be included in the study under the condition that they suffered from type 2 DM treated with metformin and provided that they were at high or exceptionally high cardiovascular risk. Poor glycemic control, defined by HbA1c > 7%, was an inclusion criterion for this study. High cardiovascular risk was defined as a ten-year risk for a cardiovascular event ≥5% and <10%, while extremely high cardiovascular risk was defined as target organ damage in a patient with DM or the coexistence of DM with a significant risk factor, like smoking, hypercholesterolemia or hypertension (cumulative ten-year risk for a cardiovascular event ≥10%). All recruited patients were treated with statins. Hypertension was characterized as blood pressure measured >140/90 mm Hg or the utilization of an antihypertensive drug.

The presence of active malignancies, active connective tissue disease, chronic kidney impairment (glomerular filtration rate <60 mL/min per 1.73 m^2^), liver insufficiency, peripheral arteriopathy and retinopathy were determined as exclusion criteria.

Concerning exclusion criteria, in 18 patients we had screening failures. Finally, 220 subjects were randomized to receive, on top of metformin, either basal insulin [20,21] or 1.8 mg of liraglutide subcutaneously once daily or 25 mg empagliflozin per os once daily or a combination of liraglutide and empagliflozin for six months. The daily dose and titration of basal insulin were determined by the American Diabetes Association and the European Association for the Study of Diabetes [22]. Liraglutide and empagliflozin were not uptitrated, and patients were treated with 1.8 mg of liraglutide daily and/or 25 mg of empagliflozin daily after randomization.

Finally, 20 subjects did not comply with the study protocol or were lost to follow-up. In the insulin group, 5 subjects were lost to follow-up; in the liraglutide group, 3 participants had poor compliance to treatment due to gastrointestinal side effects and 1 subject was lost to follow-up; in the empagliflozin group, 4 participants had poor compliance to treatment due to urinary tract infections and 1 subject was lost to follow-up; in the combination therapy group, 3 subjects stopped due to intense gastrointestinal symptoms, 1 subject stopped because of a urinary tract infection and 2 subjects were lost to follow-up.

In total, 200 patients were included in the final analysis. In all participants, we performed clinical, vascular and echocardiographic assessments at baseline and 6 months post-treatment. Two consultant cardiologists blinded to patients’ data evaluated the echocardiographic and vascular data. The study was started in November of 2017 and patient recruitment lasted almost 18 months, and the study was carried out within 24 months, in December of 2019. Data presented are part of a larger scale study, registered in clinical trial.gov as NCT03878706 on 2 November 2019.

The research protocol received approval from the Ethics Committee of the University General Hospital “Attikon”, and all participants in the study provided written informed consent. The study was carried out in adherence to the principles outlined in the Declaration of Helsinki.

The primary end point was to investigate the alteration in left atrial function, as assessed by the LA strain after 6 months of treatment with GLP1RA (liraglutide), SGLT2i (empagliflozin), their combination and insulin.

The secondary end point was to investigate whether the alteration in left atrial function is associated with changes in arterial elastic properties and left ventricular function. Arterial elastic properties are determined by measuring pulse wave velocity, while myocardial function is assessed by the left ventricular global longitudinal strain.

## 3. Measurements

### 3.1. Blood Pressure Measurement

The recruited subjects stayed for 10 min in a quiet room to relax. An automated digital oscillometric sphygmomanometer was used for the assessment of brachial blood pressure and heart rate in both arms (TensioMed, Budapest, Hungary). Three sequential measurements were performed, while three-minute intervals mediated between measurements were obtained and the mean value was used for a statistical analysis [23].

### 3.2. Central Hemodynamics

We measured the carotid-femoral PWV and central aortic pressures (central systolic and diastolic) using tonometry by Complior (Alam Medical, Vincennes, France). The measurement of cf-PWV is a noninvasive method that provides information about arterial stiffness. Arterial stiffness is an important parameter that reflects the elasticity of the arteries. The assessment of carotid femoral pulse wave velocity using a Complior device Alam Medical, Vincennes, France). typically involves the use of sensors and algorithms to calculate the time taken for the pulse wave to travel between the carotid and femoral arteries. The patient is usually asked to rest in a supine position for about 10–15 min before the assessment. The procedure generally involves placing pressure sensors on these arteries and recording the pressure waveforms. The device then calculates the time delay between the pulse reaching the carotid and femoral arteries, and after inserting the measurement of distance between the carotid and femoral artery, the pulse wave velocity is derived. The increased PWV is associated with increased arterial stiffness, which is a risk factor for adverse cardiovascular events. Normal values are PWV < 10 m/s [24]. For the calculation of cf-PWV, we used the direct method for the measurement of the distance between the carotid and femoral artery (Complior; Alam Medical, Vincennes, France), and the appropriate corrections of PWV were calculated by multiplying the default PWV measurement by 0.8.

### 3.3. Echocardiography

Examinations were conducted utilizing a Vivid E95 ultrasound system from GE Medical Systems in Horten, Norway. The digital storage of the studies was carried out on a computerized station (EchoPac GE202, Horten, Norway). Two observers, unaware of clinical and laboratory data, independently analyzed all of the studies.

We assessed the global longitudinal strain (GLS %) of the left ventricle (LV) using 2-dimensional echocardiography images captured at a frame rate ranging from 60/s to 80/s. The images were obtained from the apical 4-, 2- and 3-chamber views, employing a 17 LV segment model and dedicated software (EchoPac PC 204; GE Healthcare, Horten, Norway) [25,26,27]. The validated normal value for GLS is −22.5 ± 2.7% [28]. The intraobserver and interobserver reproducibility values for the LV strain parameters were determined as 8% and 9%, respectively.

The LA strain (LAS) was measured by 2-dimensional echocardiography images captured at a frame rate ranging from 60/s to 80/s, from the apical 4- and 2-chamber views focused on LA by using dedicated software (EchoPac PC 204; GE Healthcare, Horten, Norway) [27].

The LAS was assessed by calculating the longitudinal deformation from all segments of the atrium in one cardiac cycle. The end of LV early filling and onset of atrial contraction were determined by the E and A Doppler waveform of the mitral inflow in 4-chamber views. The ECG can only be used as a rough estimate of different phases of the cardiac cycle. Otherwise, by observing the shape of the LA strain curve, we defined the different phases of atrial function during the cardiac cycle. LA deformation involves three distinct phases, with strain values being denoted as positive or negative based on whether the atrium lengthens or shortens during each phase: (a) reservoir phase: initiated at the conclusion of the ventricular diastole (mitral valve closure), extending until mitral valve opening. This phase encompasses LV isovolumic contraction, ejection and isovolumic relaxation. (b) Conduit phase: extends from the moment of mitral valve opening through diastasis until the initiation of LA contraction in patients with a sinus rhythm. For individuals with atrial fibrillation, this phase persists until the termination of the ventricular diastole (mitral valve closure). (c) Contraction phase: takes place from the onset of LA contraction until the ending of the ventricular diastole (mitral valve closure) in patients with a sinus rhythm. The measurement reference point is contingent on the defined zero point, and in this context, we designated LV end-diastole as the zero point for our measurements [27].

## 4. Statistical Analysis

Regarding the statistical power calculation of the study, we conducted an initial pilot study. The analysis was carried out with dedicated software PASS 2024, v24.0.1 (NCSS, Kaysville, UT, USA)as previously described [17].

We employed SPSS 22.0 software (SPSS Inc., Chicago, IL, USA) for the data analysis. Mean values are presented with standard deviation (SD), while categorical variables are displayed as percentages. The normal distribution of continuous variables was assessed using the Kolmogorov–Smirnov test. Categorical data were analyzed using the χ^2^ test.

All analyses were conducted on an intention-to-treat basis. The ANOVA (general linear model; SPSS 22; SPSS Inc.) was utilized for analyzing repeated measurements. This involved examining markers both at baseline and 6 months after treatment, treated as a within-subject factor, and assessing treatment effects as a between-subject factor (including insulin, liraglutide, empagliflozin and combination liraglutide and empagliflozin). The interaction between the time of measurement for markers and the examined covariates was evaluated, along with comparisons between treatments, with F and *p* values calculated. The Greenhouse–Geisser correction was applied if the assumption of sphericity, as determined by Mauchly’s test, was violated. Post-hoc comparisons were adjusted using Bonferroni’s correction. Additionally, the ANOVA was used to analyze the percentage changes in examined variables post-treatment between study groups. All statistical tests were two-tailed, with a significance level set at *p* < 0.05.

## 5. Results

We studied 200 patients with type II DM at high or very high risk for cardiovascular complications (59.5 ± 9.1 year old, 151 male). The baseline characteristics, risk factors and treatment are described in Table 1.

Compared to baseline, the LA reservoir strain remained unchanged 6 months post-treatment in patients treated with insulin (33 ± 8.3% vs. 32.8 ± 7.4%, Δ%= −3.13 ± 1.2%, *p* = 0.829). Conversely, the LA reservoir strain increased after treatment in patients treated with liraglutide (30.7 ± 9.3% vs. 33.9 ± 9.7%, Δ% = 7.9 ± 2.1%, *p* = 0.011), empagliflozin (30 ± 8.3% vs. 32.3 ± 7.3%, Δ% = 8.2 ± 2.2%, *p* = 0.040) and the combination of empagliflozin and liraglutide (29.1 ± 8.7% vs. 31.3 ± 8.2%, Δ% = 10.1 ± 1.9%, *p* = 0.007) (Table 2). A significant interaction between the changes in the LA reservoir strain and type of treatment was observed (P for interaction = 0.002). At 6 months, compared with the other three treatment groups, the combination resulted in a greater improvement in the LA reservoir strain (10.1% versus −3.13% in insulin, 7.9% in liraglutide and 8.2% in empagliflozin group; *p* = 0.005, *p* = 0.015 and *p* = 0.017).

Compared to baseline, the LA conduit strain remained unchanged post-treatment in patients treated with insulin (−16.2 ± 5.5% vs. −15.9 ± 4.5%, *p* = 0.516), and in the empagliflozin group there was a nonsignificant decrease (−13.8 ± 4.8% vs. −14.9 ± 4.4%, *p* = 0.216) (Table 2).

Conversely, the LA conduit strain increased after treatment with liraglutide (−14.3 ± 6.1% vs. −16.1 ± 5.9%, *p* = 0.039) and the combination of empagliflozin and liraglutide (−12.9 ± 4.2% vs. −14.5 ±.2%, *p* = 0.010).

No significant changes were noted for the LA contractile strain throughout the study in the different categories (*p* > 0.05) (Table 2).

Compared to baseline, E/e’ remained unchanged post-treatment in patients treated with insulin (9.7 ± 3.3 vs. 9.4 ± 2.5, *p* = 0.235). Conversely, E/e’ decreased after treatment with liraglutide (9.1 ± 2.7 vs. 7.9 ± 2.5, *p* = 0.032), empagliflozin (9.3 ± 3.1 vs. 7.5 ± 2.6) and the combination of empagliflozin and liraglutide (9.1 ± 2.7 vs. 7.2 ± ±2.4, *p* = 0.005) (Table 2).

Compared to baseline, GLS remained unchanged after treatment with insulin (−18.5 ± 3.9% vs. −18.9 ± 3.5%, *p* = 0.202), while in the empagliflozin group, in the liraglutide group and in the combination group there was a significant improvement [(−18.1 ± 3.9% vs. −19.1 ± 3.8%, *p* = 0.039), (−18.2 ± 4.1% vs. −19.1 ± 3.5%, *p* = 0.03), (−16.7 ± 3.9% vs. −17.5 ± 4.3%, *p* = 0.026), respectively] (Table 3).

Compared to baseline, PWV did not alter in the insulin group (12.06 ± 3.02 vs. 11.62 ± 2.79 m/s, *p* = 0.142), while a significant decrease was noticed in the liraglutide group (11.54 ± 2.91 vs. 11.05 ± 2.52 m/s, *p* = 0.032). Also, in the empagliflozin and in the combination of empagliflozin and liraglutide groups there were significant reductions in PWV [(11.86 ± 2.51 vs. 11.33 ± 2.28 m/s, *p* = 0.047), (12.01 ± 2.46 m/s vs. 10.85 ± 1.79 m/s, *p* = 0.021), respectively] (Table 3).

Compared to baseline, there was a significant reduction in HbA1c in the insulin group, in the liraglutide group, in the empagliflozin group and in the combination of empagliflozin and liraglutide [(8.1 ± 1.1 vs. 6.9 ± 1.0, *p* = 0.019), (8.1 ± 0.9 vs. 7.0 ± 0.9, *p* = 0.017), (7.9 ± 0.9 vs. 7.1 ± 1, *p* = 0.035), (8.0 ± 1.2 vs. 6.4 ± 0.8, *p* = 0.006), respectively] (Table 3).

### Correlations

At baseline, there was a correlation of the LA reservoir strain with PWV (r = −0.209, *p* = 0.008), central SBP (r = −0.151, *p* = 0.030), EF (r = 0.214, *p* = 0.004) and GLS (r = −0.279, *p* = 0.009).

The LA reservoir change 6 months post-treatment was correlated with the PWV change in all groups (r = −0.242, *p* = 0.028).

The LA reservoir change 6 months post-treatment was correlated with the GLS change in all groups (r = −0.322, *p* = 0.004).

## 6. Discussion

In our study, we showed that treatment with liraglutide and empagliflozin for 6 months led to a greater improvement in the left atrial function compared to insulin treatment, despite a similar glycosylated hemoglobin reduction. Particularly, this improvement was more evident in patients treated with the combination of liraglutide and empagliflozin, as shown by a greater percental change in the LA reservoir strain in the combination group compared to the other treatment groups. Moreover, the beneficial effects of liraglutide and empagliflozin on left atrial function were in parallel with the improvement in arterial and left ventricular function, as assessed by PWV, E/e’ and LV myocardial deformation, respectively.

SGLT-2i (empagliflozin) contribute to blood pressure decrease and increase diuresis, because of their natriuretic and glucosuretic action. Furthermore, these drugs hinder ultrafiltration from the kidneys and provide long-term renal protection [8]. Their action hampers volume overload and contributes to the maintenance of the euvolemic status of patients with diabetes. As a result, the left ventricular end diastolic pressure is reduced, left ventricular compliance is improved and atrioventricular coupling is enhanced as the left atrium has a lower load to encounter. In line with this hypothesis, we have found an improved LA strain related to the improvement in LV myocardial deformation.

The above effects on LA deformation may provide an additional, pathophysiological mechanism supporting the beneficial effects of empagliflozin on cardiovascular events in HFrEF and HFpEF patients, regardless of the presence of DM [8,29].

Moreover SGLT-2i improve the energy metabolism of the myocardial cell, inhibit inflammation and decrease oxidative stress as previously shown [30,31]. Their action enhances the improvement of autophagy and lysosomal degradation and decreases SGLT-1 activity. Furthermore, epicardial fat mass is diminished and cardiac remodeling is hindered. More of these mechanisms are also speculated to improve vascular function. Moreover, aiding in weight loss, increasing erythropoietin production and diminishing sympathetic system action have additive value to improve myocardial function [31], as shown by the improved LA and LV strain post-empagliflozin treatment in the current study.

GLP-1RA have cardiovascular benefits as they ameliorate glucose metabolism and reduce insulin resistance. Treatment with GLP1RA results in significant weight loss by reducing visceral adipose tissue. Visceral adipose tissue stimulates insulin resistance, by increasing insulin and proinsulin excretion from the beta cell. Unphysiological levels of insulin and proinsulin promote atherogenesis through the activation of endothelial mitogen-activated protein kinase. Preadipocytes secrete numerous cytokines involved in the pathogenesis of hypertension, dyslipidemia and the firing of inflammation. As a result, GLP-1RA treatment contributes to an improvement in lipid metabolism, to a reduction of blood pressure and to an improvement in arterial elastic properties, as shown by the reduction of central systolic blood pressure in our study [32].

Previous observations in mice models of advanced macrovascular disease have claimed that treatment with the GLP-1R agonist liraglutide conferred attenuation of atherosclerosis and plaque stabilization as well as reduced vascular inflammation and oxidative stress [33,34,35]. Contemporary investigation supports the potential for liraglutide and empagliflozin to attenuate cardiac fibrosis in multiple pathological settings identified in both diabetic and nondiabetic states and scrutinize the molecular mechanisms associated with these effects [36,37]. The above mechanism may support a direct effect of GLP1 treatment to LA myocardium leading to an improved LA strain, as revealed in our study.

Moreover, GLP-1RA hinder atherosclerosis progression, diminish inflammation burden and protect endothelial function [10,38,39]. Thus, GLP-1RA are approved medications prescribed on top of metformin in diabetics with atherosclerotic disease. Furthermore, it is proposed that GLP-1RA are also beneficial in patients with heart failure, when an SGLT-2 inhibitor cannot be prescribed or is insufficient for glucose control [40].

In a previous study [17], we have shown that twelve months post-treatment with GLP-1RA (liraglutide), SGLT-2i (empagliflozin) or their combination, all patients had improved global longitudinal strain, global circumferential strain and global radial strain compared with patients treated with insulin, and patients treated with liraglutide or a combination of liraglutide and empagliflozin provided a greater increase in the global work index compared with insulin or empagliflozin. We have now extended these findings by showing that an improvement in arterial function and LV is linked to an improved LA stain, a surrogate marker reflecting LA and LV filling pressures.

In the current study, we have shown that treatment with empagliflozin or liraglutide alone or combined reduced arterial stiffness, as assessed by PWV. Both and their combination had an effect on the central systolic BP, as seen in Table 3. Our findings suggest that the combined administration of empagliflozin with liraglutide has a more pronounced effect on arterial wall properties than each substance separately. Experimental studies support that GLP-1RA may be effective for the treatment of hypertension in mice [33]. Studies also support that both GLP1 and SGLT2 may improve endothelial function [16,32,34]. Thus, both novel antidiabetic regimens may reduce the LV afterload, resulting in improved cardiac and atrial function.

By improving, simultaneously, the cardiac and vascular function, ventriculo–arterial coupling is facilitated, and myocardial reserve is spared. A simplified index of ventriculo–arterial interaction is expressed by the ratio PWV/GLS [18]. According to our study, both vascular and cardiac function markers were improved after 6 months treatment with novel antidiabetic treatment, resulting in a decline of left ventricular end-diastolic pressure, better ventricular compliance and thus an improvement in the left atrial strain. Indeed, in our study there was a significant reduction of E/E’ after treatment with novel antidiabetic medications compared to insulin, indicating a reduction in LV diastolic filling pressures.

Additionally, experimental studies support that both SGLT-2 and GLP1RA hinder the development of fibrosis in myocardium [36,37]. Beyond the antifibrotic effect that they have in the liver, in which they reduce hepatic fibrosis and steatosis, these regimens seem to attenuate fibrosis formation in other vital organs, such as the heart, the kidney and the lung. This action, in addition to endothelial protection, inflammation suppression and oxidative stress reduction prevent fibrosis and improve atrial function [36,37].

According to a recent metanalysis including trials with available GLP1 RA and SGLT2i specimens, it is claimed that SGLT2i and GLP1-RA demonstrate a comparable reduction in MACE among patients with documented cardiovascular disease. However, in patients without cardiovascular complications, neither SGLT2i nor GLP1-RA exhibits a noticeable effect on MACE over the documented timeframe. Notably, SGLT2i, unlike GLP1-RA, decreases the risk of heart failure development. Regarding kidney function, GLP1-RA lowers the risk of macroalbuminuria, while SGLT2i hinders renal function deterioration (8). Moreover, regarding nondiabetic patients, there is only evidence for treatment with SGLT2i in patients with HFpEF/HFrEF [41]. According to a recent metanalysis, in nondiabetic obese individuals, liraglutide was found to decrease body weight, BMI and blood pressure compared to the placebo. Adverse events, Hb1Ac levels and hypoglycemic episodes did not differ significantly from those in the placebo group [42]. Additionally, researchers in the selected study observed that in patients with pre-existing cardiovascular disease who were overweight or obese but not diabetic, weekly subcutaneous semaglutide outperformed the placebo in reducing the occurrence of death from cardiovascular causes, nonfatal myocardial infarction or nonfatal stroke over an average follow-up period of 39.8 months [43].

## 7. Limitations

This is a single-center study with a short follow-up period. Further investigation is needed in order to detect whether these findings persist after longer time periods and whether these findings occur for other GLP1RA and other SGLT2i. Furthermore, the LA strain measurements have a significant variation when different vendors are used. The two-dimensional strain is influenced by the quality of the image, resulting in limitations due to dropout regions, notably in the left atrium roof and the confluence of pulmonary veins. Additionally, the atrium being in the far field contributes to these limitations. Nevertheless, there is now specialized software designed for assessing left atrial strain, which holds promise in addressing the variability encountered in these measurements.

## 8. Conclusions

Treatment with liraglutide, empagliflozin and their combination for 6 months showed a greater improvement in left atrial function as assessed by the left atrial strain compared to insulin treatment in parallel with an improvement in arterial and myocardial function.

## Figures and Tables

**Table 1:** Demographic and clinical characteristics of participants in each of the study groups.

	All (N = 200)	Insulin (N = 50)	GLP1 (Liraglutide) (N = 50)	SGLT2 (Empagliflozin) (N = 50)	GLP1 & SGLT2 (Liraglutide and Empagliflozin) (N = 50)	*p*
AGE (YEARS)	59.5 ± 9.1	60.5 ± 9.1	58.1 ± 8.8	59.2 ± 9.2	59.3 ± 8.9	0.615
SEX (MALE)	161 (80.5%)	37 (74%)	44 (88%)	38 (76%)	42 (84%)	0.232
DM DURATION (YEARS)	8.1 ± 9.1	7.2 ± 7.1	5.6 ± 6.2	9.1 ± 6.7	9.6 ± 6.8	0.039
BMI (kg/m^2^)	32.2 ± 5.9	30.1 ± 5.9	34.7 ± 6.4	30.9 ± 5.4	34.4 ± 5.2	<0.001
HFpEF	46 (23%)	12 (24%)	11 (22%)	10 (20%)	13 (26%)	0.565
HFrEF	16 (8%)	5 (10%)	4 (8%)	3 (6%)	4 (8%)	0.675
FAMILY HISTORY	85 (42.5%)	23 (46%)	17 (34%)	22 (44%)	23 (46%)	0.362
SMOKING	90 (45%)	30 (60%)	19 (38%)	21 (42%)	20 (40%)	0.630
AMI	82 (41%)	19 (38%)	12 (24%)	27 (54%)	24 (48%)	0.002
HYPERTENSION	139 (69.5%)	40 (80%)	25 (50%)	30 (60%)	44 (88%)	0.159
HYPERLIPIDEMIA	142 (71%)	35 (70%)	29 (58%)	38 (76%)	40 (80%)	0.508
Hypertension Drugs	139 (69.5%)	37 (74%)	21 (42%)	35 (70%)	46 (92%)	0.095
CCB	59 (29.5%)	15 (30%)	9 (18%)	15 (30%)	20 (40%)	0.172
ACEi/ARBs	137 (68.5%)	36 (72%)	25 (50%)	34 (68%)	42 (84%)	0.06
b-blocker	103 (51.5%)	27 (54%)	13 (26%)	34 (68%)	29 (58%)	0.001
diuretic	54 (27%)	18 (36%)	12 (24%)	12 (24%)	12 (24%)	0.941
Aldosterone inhibitor	19 (9.5%)	4 (8%)	5 (10%)	3 (6%)	7 (14%)	0.230
statin	169 (84.5%)	42 (84%)	40 (80%)	46 (92%)	41 (82%)	0.832
fibrates	25 (12.5%)	3 (6%)	5 (10%)	8 (16%)	9 (18%)	0.373

ACEi: Angiotensin-converting enzyme inhibitors; AMI: history of acute myocardial infarction; ARBs: Angiotensin receptor blockers; BMI: body mass index; CCB: calcium channel blockers; HFpEF: heart failure preserved ejection fraction; HFrEF: heart failure reduced ejection fraction.

**Table 2:** Left atrial deformation parameters in the four study groups at baseline and 6 months post-treatment.

	Total	Insulin	GLP1 (Liraglutide)	SGLT2 (Empagliflozin)	GLP1 & SGLT2 (Liraglutide and Empagliflozin)
LASr baseline (%)	31.2 ± 8.8	33 ± 8.3	30.7 ± 9.3	30 ± 8.3	29.1 ± 8.7
LASr follow-up (%)	32.4 ± 8.1	32.8 ± 7.4	33.9 ± 9.7 *	32.3 ± 7.3 *	31.3 ± 8.2
*p*	0.005	0.829	0.011	0.040	0.007
LAScd baseline (%)	−14.4 ± 5.2	−16.2 ± 5.5	−14.3 ± 6.1	−13.8 ± 4.8 ^*^	*
LAScd follow-up (%)	−15.3 ± 4.6	−15.9 ± 4.5	−16.1 ± 5.9 *	−14.9 ± 4.4	−14.5 ± ±4.2
*p*	0.015	0.516	0.039	0.216	0.010
LASct baseline (%)	−16.9 ± 5.3	−17 ± 5	−17.1 ± 5.6	−16.8 ± 5.1	−16.8 ± 5.7
LASct follow-up (%)	−17.4 ± 4.8	−16.6 ± 4.6	−18 ± 5.1 *	−18.1 ± 4.5 *	−17.3 ± 5.3
*p*	0.175	0.449	0.298	0.118	0.475
E/e’ baseline	9.2 ± 3.4	9.7 ± 3.3	9.1 ± 2.7	9.3 ± 3.1	9.1 ± 2.7
E/e’ follow-up	8.5 ± 2.8	9.4 ± 2.5	7.9 ± 2.5 *	7.5 ± 2.6 *	7.2 ± 2.4 *
*p*	0.055	0.235	0.032	0.011	0.005

Data are presented as mean ± SD. LASr: left atrial strain during atrial reservoir phase; LAScd: left atrial strain during atrial conduit phase; left atrial strain during atrial contraction phase; E: early mitral inflow velocity by Doppler; e’: average lateral and septal velocity of mitral annulus by tissue doppler. * *p* < 0.05, for time × treatment interaction obtained by repeated-measures ANOVA. P presented in Table reflects comparisons of 6 months vs. baseline by ANOVA using post-hoc analysis with Bonferroni correction.

**Table 3:** Arterial stiffness, left ventricular deformation parameters and glycated hemoglobin in the four study groups at baseline and 6 months post-treatment.

	Total	Insulin	GLP1 (Liraglutide)	SGLT2 (Empagliflozin)	GLP1 & SGLT2 (Liraglutide and Empagliflozin)
PWV baseline (m/s)	11.98 ± 2.76	12.06 ± 3.02	11.54 ± 2.91	11.86 ± 2.51	12.01 ± 2.46
PWV follow-up (m/s)	11.56 ± 2.47	11.62 ± 2.79 *	11.05 ± 2.52 *	11.33 ± 2.28 *	10.85 ± 1.79 *
*p*	0.008	0.142	0.032	0.047	0.021
SBP CENTRAL baseline (mmHg)	127.8 ± 18.3	130.8 ± 19.2	120.6 ± 14.6	125.8 ± 18.7	130.6 ± 18.5
SBP CENTRAL follow-up (mmHg)	125.6 ± 18.9	131.2 ± 19.8	119.2 ± 15.4 *	122.3 ± 16.2 *	127.1 ± 19.8 *
*p*	0.349	0.987	0.045	0.023	0.010
GLS baseline (%)	−17.9 ± 4.1	−18.5 ± 3.9	−18.2 ± 4.1	−18.1 ± 3.9	−16.7 ± 3.9
GLS follow-up (%)	−18.5 ± 3.9	−18.9 ± 3.5	−19.1 ± 3.5 *	−19.1 ± 3.8 *	−17.5 ± 4.3 *
*p*	0.026	0.202	0.03	0.039	0.026
HbA1c (%) baseline	8.0 ± 1.2	8.1 ± 1.1	8.1 ± 0.9	7.9 ± 0.9	8.0 ± 1.2
HbA1c (%) follow-up	6.7 ± 1.1	6.9 ± 1.0 *	7.0 ± 0.9 *	7.1 ± 1 *	6.4 ± 0.8 *
*p*	0.015	0.019	0.017	0.035	0.006

Data are presented as mean ± SD. PWV: pulse wave velocity; SBP: systolic blood pressure; GLS: global longitudinal strain by speckle tracking echocardiography; HbA1c: glycated hemoglobin. * *p* < 0.05, for time × treatment interaction obtained by repeated-measures ANOVA. P presented in Table reflects comparisons of 6 months vs. baseline by ANOVA using post-hoc analysis with Bonferroni correction.

## Data Availability

Data are available upon reasonable request.
